# Primitive visual channels have a causal role in cognitive transfer

**DOI:** 10.1038/s41598-021-88271-y

**Published:** 2021-04-22

**Authors:** William Saban, Gal Raz, Roland H. Grabner, Shai Gabay, Roi Cohen Kadosh

**Affiliations:** 1 Department of Psychology, IIPDM, University of Haifa, Haifa, Israel; 2 Department of Experimental Psychology, Wellcome Centre for Integrative Neuroimaging, University of Oxford, Oxford, UK; 3 Institute of Psychology, University of Graz, Graz, Austria; 4 Department of Psychology, Helen Wills Neuroscience Institute, University of California, Berkeley, CA USA

**Keywords:** Neuroscience, Cognitive neuroscience, Psychology, Human behaviour

## Abstract

Scientific investigations have long emphasized the cortex’s role in cognitive transfer and arithmetic abilities. To date, however, this assumption has not been thoroughly empirically investigated. Here we demonstrated that primitive mechanisms—lower visual channels—have a causal role in cognitive transfer of complex skills such as symbolic arithmetic. We found that exposing only one monocular channel to a visuospatial training resulted in a larger transfer effect in the trained monocular channel compared to the untrained monocular channel. Such cognitive transfer was found for both novel figural-spatial problems (near transfer) and novel subtraction problems (far transfer). Importantly, the benefits of the trained eye were not observed in old problems and in other tasks that did not involve visuospatial abilities (the Stroop task, a multiplication task). These results challenge the exclusive role of the cortex in cognitive transfer and complex arithmetic. In addition, the results suggest a new mechanism for the emergence of cognitive skills, that could be shared across different species.

## Introduction

Being able to learn new skills and to transfer them to new materials is essential across the lifespan and in different settings such as the educational system, the workplace, and rehabilitation. Transfer of skills (henceforth, transfer) allows us to generalize skills from one context to another, and thus has important basic and translational implications^1, 2^. Thus, transfer has attracted massive attention in the fields of psychology^3^, education^4^, neuroscience^5^, medicine^6^, and artificial intelligence^7^, and has been a topic of major debate for over 100 years^8, 9^. Consequently, the reasons for successful or unsuccessful transfer are still not fully understood^9, 10^. Notably, the ability to transfer is not limited to humans, but is also exhibited by other animals, including insects^11^, which do not have the same cortical brain networks that are assumed to support transfer in humans. It is possible that in some non-human species more primitive, non-cortical, regions are involved in transfer processes. The underrepresentation of such non-cortical regions in the neuroscience literature may be the result of a “corticocentric bias” by which the role of cortical regions in cognition is overemphasized^12^. Recently, many high-level cognitive functions were found to be based on evolutionarily ancient brain circuits. In contrast to the traditional perspective, low-level neural mechanisms (e.g., subcortical regions) were demonstrated to also have a functional role in high-level cognitive processes, such as volitional attention, temporal expectancy and numerical abilities, including subtraction^13–21^.

Here we examined whether primitive low-level visual channels, which are shared across species, may have a causal role in transfer processes. This question is rooted in recent theories that suggest that neural circuitry can be recycled and tuned for different purposes either phylo- or ontogenetically^22, 23^. Therefore, we hypothesized that during evolution the association cortex took control of subcortical and sensory cortical mechanisms and, in a dynamic interaction, enabled transfer processes. Brain architectural organization is subject to strong anatomical and connectional constraints inherited from evolution, and to develop new abilities, the brain has to find a “neuronal niche,” a set of circuits that are sufficiently close to the required function ^23^. The involvement of common primitive neural mechanisms both in basic perceptual processes and high-level cognitive processes may predict a close neural and functional link between them. Hence, if an evolutionarily older function, such as spatial perception, is used for the development of a novel one, such as subtraction, prior neural constraints may exert a powerful influence on brain organization. Furthermore, because even organisms that possess only rudimentary neural systems have the ability to transfer skills^11^, ubiquitous neural systems (UNS), which are shared across species, might be involved in these processes. These theoretical frameworks and converging empirical evidence fuel the motivation to examine whether primitive low-level visual channels are functionally involved in transfer processes.

**Figure 1:**
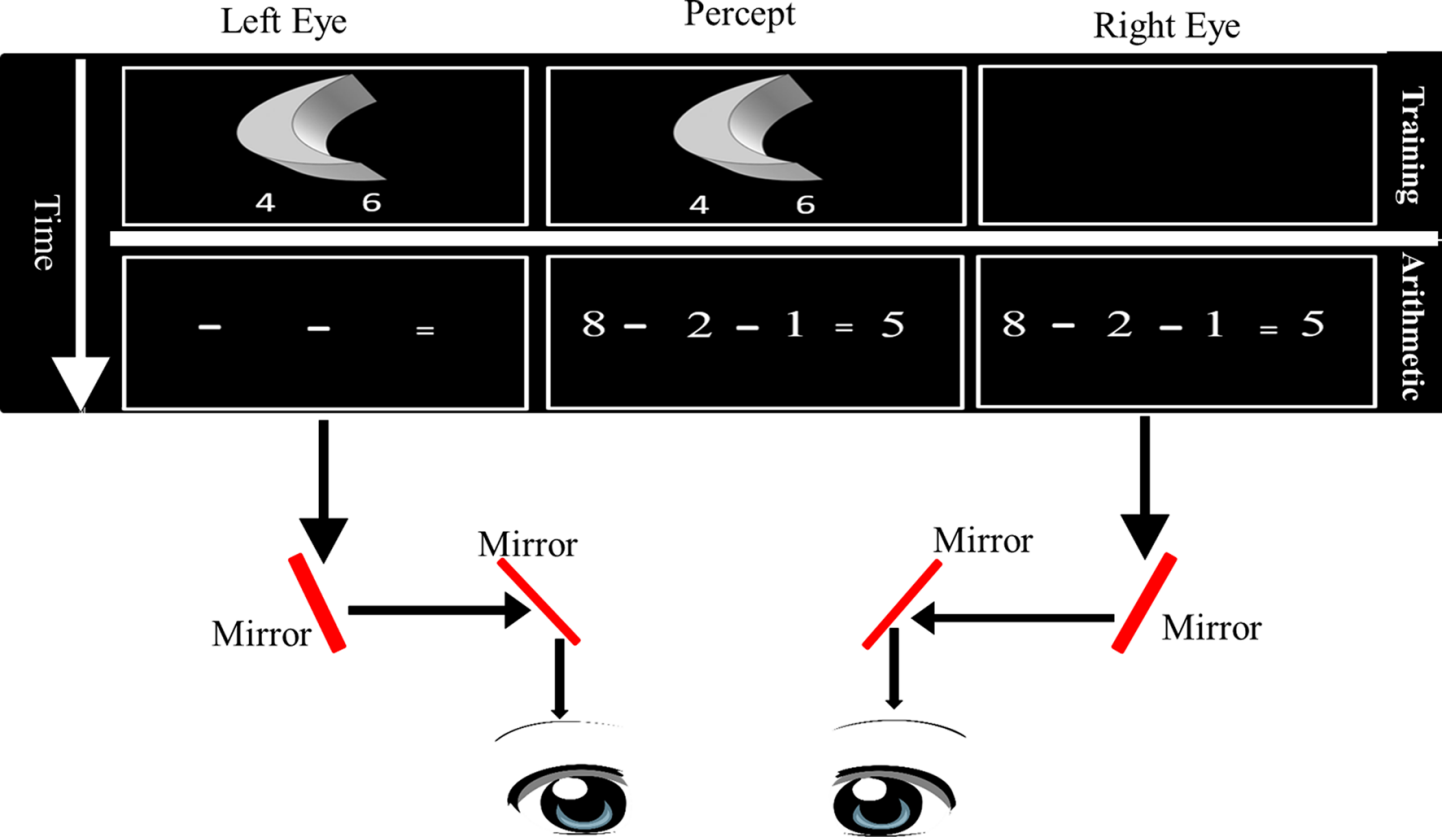
Schematic illustration of the experimental apparatus. The visual information passes from the screen monitor—through the eyes—to the brain. Each side of the computer monitor provided visual information to a different eye. From the eye, the visual information passes first through monocularly segregated subcortical regions. This information is then projected to the lateral geniculate nucleus (LGN) and subsequently reaches striate and binocular extrastriate regions.

To examine the functional contribution of primitive low-level visual channels in cognitive transfer in the mature human brain we used a stereoscope—a non-intrusive psychophysical method—to dissociate between higher (association cortex) and lower (subcortical and primary visual cortex) visual channels in order to examine their involvement in a cognitive process^15–18, 24, 25^(see Fig. 1). Visual input is monocularly segregated until it reaches striate and extrastriate regions^26, 27^. Thus, subcortical visual pathways are monocularly segregated, while higher cortical regions are mostly insensitive to the stimuli’s eye-of-origin. By dividing the visual input between the eyes, we manipulated the information processing in subcortical brain areas and V1. Notably, previous studies which utilized this stereoscopic manipulation demonstrated that monocular perceptual training effects could be restricted to the specific trained eye^28, 29^. Note, it is not possible to claim that the subcortex has an exclusive causal role in cognitive processes using the stereoscopic manipulation. A limitation with other methods (e.g., functional magnetic resonance imaging (fMRI) and EEG) is that they are fundamentally correlational, revealing the potential relationship between cognitive events and brain regions. However, the current eye-of-origin manipulation is an approach that provides a test of causality compared to other methods. Since the stereoscope device allows the manipulation of visual information to different eyes, the comparison between the two Eye conditions allows one to infer a functional role. To examine whether monocular channels are involved in cognitive transfer processes, we presented the visual information to only one monocular channel (one eye) during cognitive training to test whether transfer would be enhanced in the trained eye (vs. the eye that was not exposed to the training task) after the training stage.

The previous theoretical view and empirical evidence suggest that transfer between abilities occurs if the trained task and the untrained task engage specific overlapping neural substrates and processing components^8, 30, 31^. Therefore, we utilized a short figural-spatial training task that was shown to result in similar neurophysiological training effects for both arithmetic and visuospatial abilities^32, 33^. It was demonstrated that one session of practice can lead to significant learning benefits^32^. In the figural-spatial training task of the current study, participants were trained on figural-spatial problems, in which they were requested to determine the number of faces of three-dimensional geometric objects. Neuroimaging and neuropsychological studies suggest that subtraction is highly related to visuospatial abilities^34–37^. While subtraction is mostly related to spatial processing, multiplication is mostly related to phonological, fact-retrieval or verbal processing^38, 39^. Hence, we predicted that training on figural-spatial problems will result in training benefits mainly in the subtraction task. If, as predicted, primitive low-level visual channels are functionally involved in transfer, exposing only one eye to a figural-spatial training task will increase the transfer effect in the trained eye compared to the untrained eye in subtraction. By contrast, a lack of difference between the trained eye and untrained eye conditions do not indicate that monocular channels have a functional role in the transfer process. A pattern in which there is no difference between the Eye conditions suggests that the locus of improvement could be in subcortical-monocular or cortical-binocular channels.

Before the training task we assessed the participants’ baseline abilities in a subtraction task and a Stroop task. During training, participants performed a figural-spatial task^32, 33^. After training, we reassessed the participants’ abilities and measured the training benefits in the figural-spatial and subtraction tasks. This design allowed us to examine participants’ performance on the same figural-spatial training stimuli (stimuli repetition), novel figural-spatial stimuli (near transfer, as these stimuli shared many features with the training stimuli^9^), and novel subtraction problems (far transfer). As opposed to ‘near transfer’, which is defined as a transfer to a more similar context to that of training, ‘far transfer’ is defined as a transfer to a dissimilar context^9^. Note that ‘far transfer’ is often viewed as a transfer between domains (e.g., spatial and arithmetic abilities). To examine whether the transfer effect is specific to subtraction we included the Stroop task—which does not involve visuospatial skills—as a control task. For each participant, only one eye (counterbalanced across participants) was exposed to the training stimuli (the “trained eye”), and performance was compared between the trained-eye condition and the untrained-eye condition in each task. In addition, in Experiment 2, we aimed to replicate the findings of Experiment 1 and to test the specificity of the effect within the arithmetic domain by examining whether the trained-eye effects occur also on multiplication.

## Experiment 1

### Results

**Figure 2:**
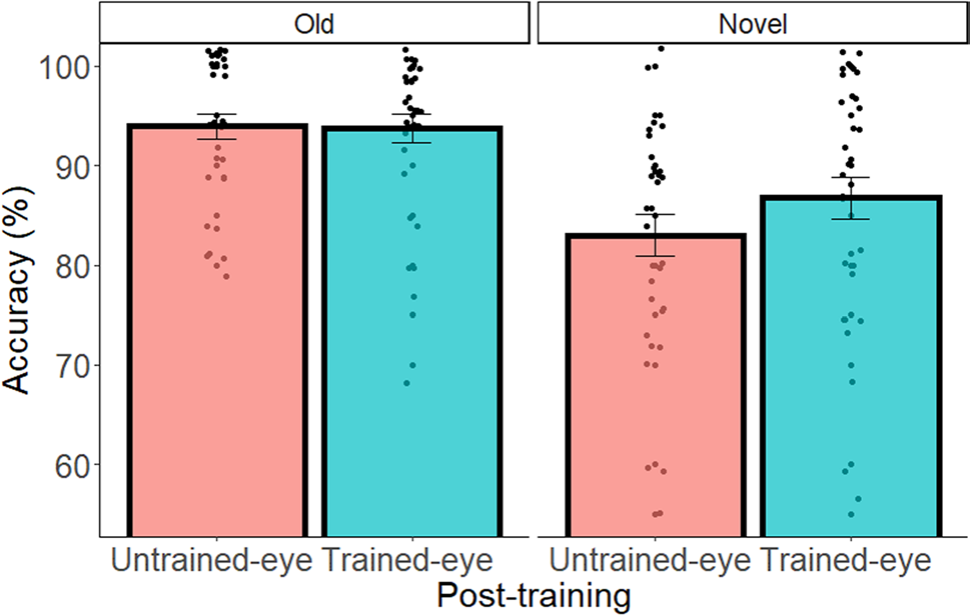
Accuracy for each individual (dot) as a function of Eye (trained-eye condition vs. untrained-eye condition) and Problem Novelty (old figures from the training task vs. novel figures) in the figural-spatial task. Error bars represent standard error of the mean (SEM). In order to compare the influence of the figural-spatial training on the trained vs. untrained eye, in the pre-training stage no figural-spatial task was presented, as in the other tasks. This was done in order to manipulate the specific influence of the figural-spatial training only on the trained eye.

We assessed the effect of training on the figural-spatial task by running two-way analyses of variance (ANOVAs) with Eye (trained eye vs. untrained eye) and Problem Novelty (old figures from the training task or novel figures) as within-subject factors, and accuracy or reaction time (RT) as the dependent variable. We observed the expected interaction between Eye and Problem Novelty on accuracy (F(1,31) = 8.02, p = 0.008, ${\upeta }_{\mathrm{p}}^{2}$=0.21), indicating a significant advantage to the trained-eye vs. untrained-eye condition for the novel but not for the old figural-spatial problems (F(1,31) = 11.16, p = 0.002, ${\upeta }_{\mathrm{p}}^{2}$=0.26; F(1,31) = 0.02, p = 0.9, ${\upeta }_{\mathrm{p}}^{2}$<0.01, respectively; for full statistical details of accuracy and RT see Table S1; see Fig. S2. for the learning curve during training). These results show that near transfer benefited more from spatial training in the trained eye compared to the untrained eye (see Fig. 2).

For the subtraction task, we carried out two-way ANOVAs with Eye (trained-eye condition vs. untrained-eye condition) and Experimental Stage (pre-training condition vs. post-training condition) as within-subject factors, and accuracy or RT as the dependent variable. The accuracy of solving novel problems after the training task was influenced by the Eye and the Experimental Stage as indicated by a significant two-way interaction (F(1,28) = 8.14, p = 0.008, ${\upeta }_{\mathrm{p}}^{2}$=0.22; for full statistical details of accuracy and RT see Table S1). This interaction was due to a significant advantage for the trained-eye condition compared to the untrained-eye condition at the post-training stage only (F(1,28) = 14.12, p < 0.001, ${\upeta }_{\mathrm{p}}^{2}$=0.30; pre-training stage: F(1,28) = 0.14, p = 0.710, ${\upeta }_{\mathrm{p}}^{2}$=0.00).

**Figure 3:**
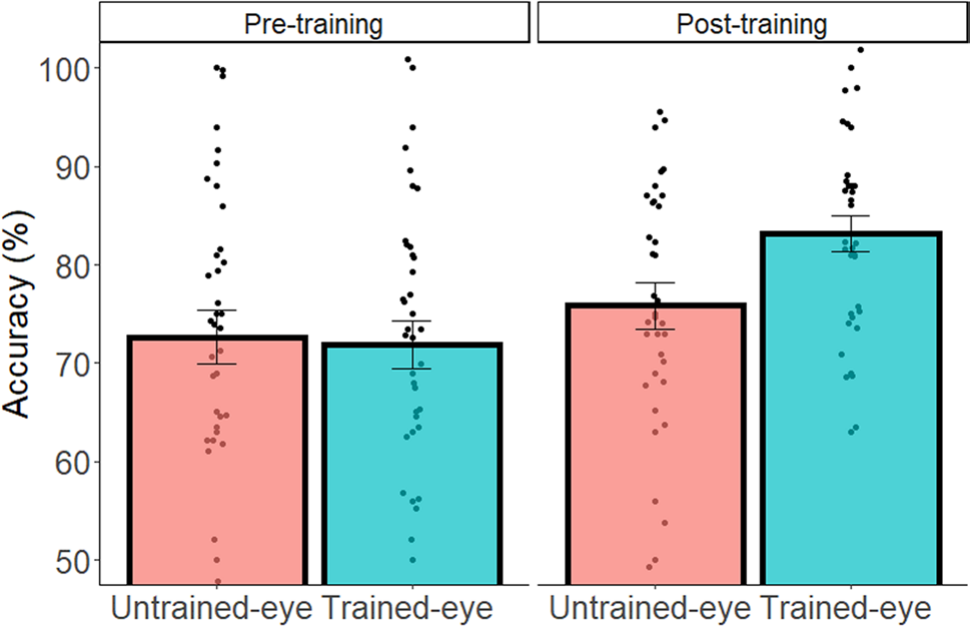
Accuracy for each individual (dot) as a function of Eye and Experimental Stage in novel subtraction problems. Error bars represent SEM.

Overall, as in the figural-spatial task, when solving novel equations, participants benefited more from spatial training in the trained-eye condition compared to the untrained-eye condition (see Fig. 3).

In contrast to these effects, the interaction between Eye and Experimental Stage was not significant for the control Stroop task (for full statistical details see Table S1).

The results of Experiment 1 demonstrate that near transfer and far transfer benefited more from primitive low-level visual channels. Both of these effects were restricted to novel problems and were not observed in problems that were presented before the post-training stage. The lack of effect in the old-problem condition reflects that it is qualitatively different from the novel-problem condition due to the likely involvement of a memory representation of the problem and of the given response, which is not dependent on the monocular channels.

## Experiment 2

In Experiment 2 we aimed to replicate these findings. Moreover, we tested the specificity of this effect within the arithmetic domain by examining whether the trained-eye transfer effects occur for subtraction, which depends mostly on spatial processing, rather than phonological, fact-retrieval or verbal memory processing in the case of multiplication^38, 39^. Aside from an additional multiplication task, Experiment 2 was similar to Experiment 1.

**Figure 4:**
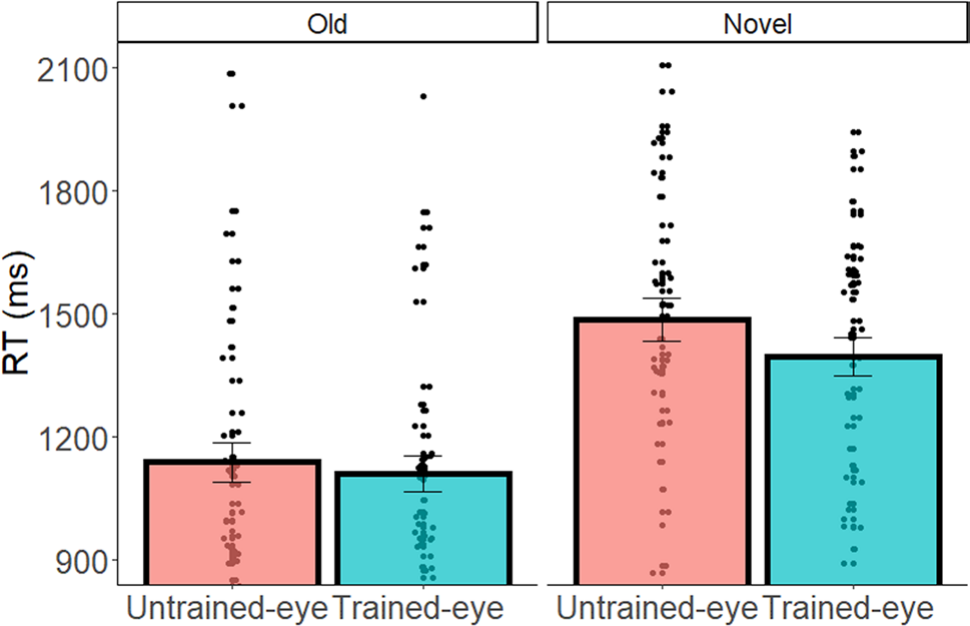
RT for each individual (dot) as a function of Eye and Problem Novelty in the figural-spatial task. Error bars represent SEM.

In the figural-spatial task, the interaction between Eye and Problem Novelty was observed only for RT (F(1,46) = 5.06, p = 0.029, ${\upeta }_{\mathrm{p}}^{2}$=0.09, Fig. 4; for full statistical details see Table S1). Follow-up planned comparisons analyses revealed a significant advantage to the trained-eye (vs. untrained-eye) condition for the novel problems, but not for the old problems (F(1,46) = 10.25, p = 0.002, ${\upeta }_{\mathrm{p}}^{2}$=0.16; F(1,46) = 1.56, p = 0.217, ${\upeta }_{\mathrm{p}}^{2}$=0.03, respectively). Note that in comparison to Experiment 1, in Experiment 2 participants also performed a multiplication task. This additional task increased the length of the experiment and might have increased the cognitive demands and fatigue. This difference may be the reason for the reduced performance in the post-training figural-spatial task of Experiment 2 compared to Experiment 1 (t(77) = 1.97, p = 0.026, 4.2% decrease in accuracy). It is possible that in this case a more sensitive measure of performance (i.e., RT) is required in order to detect the trained eye transfer effects. In addition, the lower accuracy rates in Experiment 2 indicates that the lack of trained eye effects for old problems, in both experiments, does not result from a ceiling effect. Moreover, the presence of the trained eye effect only for novel problems also in the subtraction task, is another indication for the specificity of monocular channels involvement. Importantly, in both experiments the findings highlight the improvement in information processing without a RT-accuracy tradeoff.

**Figure 5:**
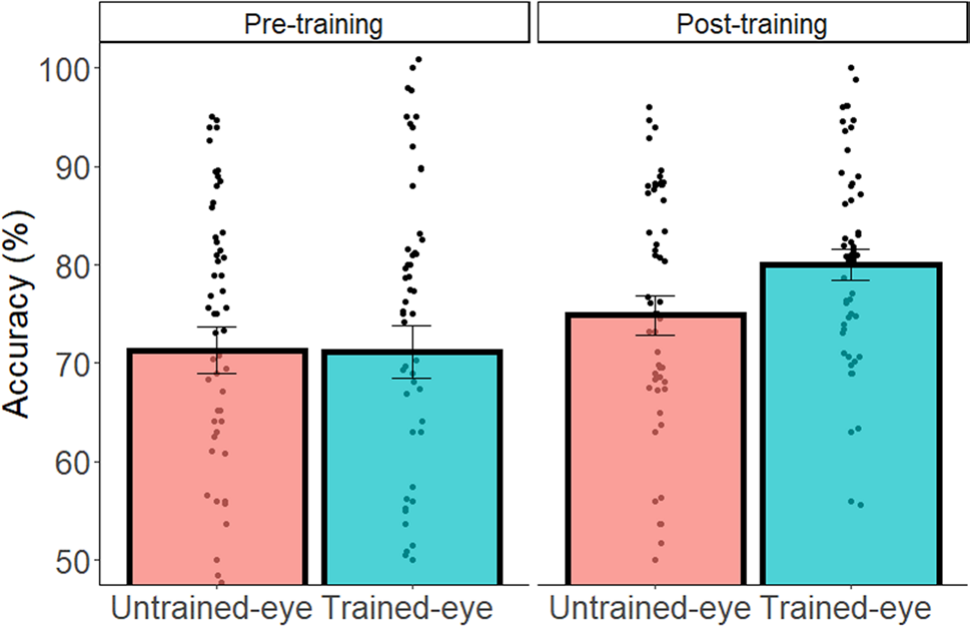
Accuracy for each individual (dot) as a function of Eye and Experimental Stage in novel subtraction problems. Error bars represent SEM.

The accuracy of solving novel problems after training was again influenced by Eye and Experimental Stage as indicated by the significant two-way interaction (F(1,45) = 4.24, p = 0.045, ${\upeta }_{\mathrm{p}}^{2}$=0.09, Fig. 5), replicating the advantage of the trained-eye (vs. untrained-eye) condition after training (F(1,45) = 8.28, p = 0.006, ${\upeta }_{\mathrm{p}}^{2}$=0.15; pre-training stage: F(1,45) = 0.02, p = 0.887,${\upeta }_{\mathrm{p}}^{2}$=0.00; see Table S1 for full statistical details of accuracy and RT; see Fig. S2 for the learning curve data during training).

Neither the multiplication task nor the Stroop task showed that training led to an eye-specific effect (see Table S1).

To examine whether the source of the observed transfer effect is rooted in cognitive changes (e.g., ability, task difficulty) effect we modeled the decision process using a diffusion decision model after training^40^. In such case we would expect that the effects observed above for subtraction and spatial-figures to be presented in the case of the drift-rate component, rather than by an auxiliary component such as encoding and response (nondecision time) or response conservativeness (boundary separation).

Both in the subtraction and spatial-figures we carried out two-way ANOVAs with Eye (trained-eye condition vs. untrained-eye condition) as within-subject factor and Experiment (Experiment 1 vs. Experiment 2) as between-subject factor, and drift-rate or encoding and response or response conservativeness as the dependent variable. In the subtraction task, the drift rate was significantly higher in the trained-eye compared to the untrained-eye condition (F(1,73) = 19.83, p < 0.001, ${\upeta }_{\mathrm{p}}^{2}$=0.214, under a direction hypothesis), and no interaction between Eye and Experiment was found (F(1,73) = 0.81, p = 0.370, ${\upeta }_{\mathrm{p}}^{2}$=0.011). Notably, this Eye effect was not observed in peripheral processes that reflect general changes in encoding and response (main effect: F(1,73) = 0.787, p = 0.378, ${\upeta }_{\mathrm{p}}^{2}$=0.011; Eye X Experiment interaction: F(1,73) = 0.28, p = 0.598, ${\upeta }_{\mathrm{p}}^{2}$=0.004) or response conservativeness (main effect: F(1,73) = 1.38, p = 0.243, ${\upeta }_{\mathrm{p}}^{2}$=0.019; Eye X Experiment interaction: F(1,73) = 0.002, p = 0.965, ${\upeta }_{\mathrm{p}}^{2}$=0.00).

Similarly, in the spatial-figures task, the drift rate effect was significant, such that it was higher in the trained-eye compared to the untrained-eye condition (main effect: F(1,77) = 3.18, p = 0.039, ${\upeta }_{\mathrm{p}}^{2}$=0.040; Eye X Experiment interaction: F(1,77) = 0.192, p = 0.662, ${\upeta }_{\mathrm{p}}^{2}$=0.002). Notably, this effect was not observed in encoding and response (main effect: F(1,77) = 0.545, p = 0.463, ${\upeta }_{\mathrm{p}}^{2}$=0.007, Eye X Experiment interaction: F(1,77) = 1.08, p = 0.301, ${\upeta }_{\mathrm{p}}^{2}$=0.014)) or response conservativeness (main effect for Eye: F(1,77) = 0.002, p = 0.965, ${\upeta }_{\mathrm{p}}^{2}$=0.00, Eye X Experiment interaction: F(1,77) = 11.34, p = 0.001, ${\upeta }_{\mathrm{p}}^{2}$=0.128).

## Discussion

These results show a monocular channel advantage when participants solved novel figural-spatial and subtraction problems after the same monocular channel was exposed to a figural-spatial training task. Importantly, these near and far transfer effects on the trained eye were not observed in the performance of other tasks that did not involve a visuospatial component, even when they included numerical information. These findings highlight that the transfer of skills to a high-level cognitive ability, such as subtraction, functionally involves primitive low-level visual channels.

The neural findings from studies examining transfer processes typically do not indicate the involvement of primitive low-level brain mechanisms in transfer^12^. The usage of fMRI in many studies, with its poor ability to detect activations in subcortical structures^41–43^, may be the origin of such a “corticocentric bias” in the neuroscience literature. Other methods, such as electroencephalography, lack the required spatial resolution. Hence, these methods for studying neural substrates of cognition are focused mainly on the association cortex and are limited in their ability to implicate the involvement of primitive brain mechanisms in cognitive processes, including transfer processes. Importantly, the current experimental manipulation allows one to draw a *causal inference* between the neural stage of processing and cognitive functioning, whereas neuroimaging methods only allow one to infer the neural correlate of transfer^19^, and transcranial magnetic and electrical stimulation methods only allow one to influence mainly cortical regions^44^.

It should be noted that perceptual differences, binocular rivalry, and intraocular suppression cannot explain the differences in performance observed between the two Eye conditions in the current experiments. First, to preclude any confounding effect of perceptual differences between the Eye conditions and to determine whether participants experienced a well-fused percept in both conditions, the stereoscope apparatus was calibrated for each participant individually to ensure perceptual fusion of the images presented in both Eye conditions (see “Methods” section for more details). Second, if only perceptual processes were involved in the trained-eye benefit, regardless of the involvement of transfer processes, performance should be better in all trained-eye conditions compared with untrained-eye conditions. This was not the case in both the Stroop task and the multiplication task. Third, the same basic perceptual factors are involved both in the old and novel problems. In both experiments, there was a difference in performance between the two eye conditions only in novel problems for subtraction and figural-spatial tasks, indicating that the stereoscope manipulation by itself cannot account for the present findings. Singly and collectively, the above-mentioned considerations render alternative, perceptual explanations for the observed differences unlikely.

In the figural-spatial training, participants need to determine the number of faces in three-dimensional geometric objects. There are two main processes involved in this training task: Spatial mental representation of the whole object and counting of the observed faces of the object. The former is a more spatial process and the latter is a more general numerical (counting abilities) process. The literature suggests that subtraction is mostly related to spatial processing, while multiplication is mostly related to phonological, fact-retravel or verbal processing^38, 39^. Training of the counting component (numerical process) can, potentially, be beneficial for both multiplication and subtraction, but the training of the spatial mental representation component is assumed to be more beneficial for subtraction. Accordingly, in the current study, the trained-eye effects were found only in subtraction but not in multiplication. Hence, theoretically, it is more reasonable that the observed trained-eye benefits are the result of training of the mental spatial representation component. However, the observed pattern of results does not allow to purely dissociate which component contribute more to the trained-eye training benefits. Future studies should examine which component (i.e., spatial or counting) contribute more to trained-eye training benefits.

Note, after prolonged exposure, the participant has a reduced need to perceptually process the minus visual sign. While perceptually there is a lower need to process the arithmetic operator sign, mental subtraction is still needed in order to solve the subtraction problems. The selectivity of the Eye effects observed only on subtraction problems, and not on multiplication problems, indicates that the effects are limited to a specific mental arithmetic operator, which are not related to the prolonged exposure to the operator visual sign that occur in both multiplication and subtraction.

The present findings extend previous views suggesting that cultural constructions, such as arithmetic, are grounded in evolutionarily ancient representations, such as space^45^. In accordance with previous literature^45^, we suggest that higher cognitive processes, such as the current human’s fully developed numerical skills, may be mediated by the recruitment of more basic processes, such as visuospatial abilities^46^. We extend this literature by showing that such skills involve primitive low-level visual channels, and not only associative cortical regions.

In recent years, evolutionarily ancient brain circuits (e.g., subcortical regions) were found to be involved in many high-level cognitive processes, such as executive functions, volitional attention, temporal expectancy and numerical abilities^13–17, 19, 20^. The findings in the present study support the notion that UNS (i.e., low-level visual channels) have a functional role in transfer processes to one of the most sophisticated human abilities, arithmetic. The results have major implications for the understanding of the neuroevolutionary development of cognitive abilities, and suggest a parsimonious explanation for the ability of different animals to transfer skills, despite their lack of similar cortical structures that have been suggested to support transfer in humans^38^. We propose that UNS may have a functional role in the development and evolution of cognition. Since UNS developed early in evolution, survived, and have remained functional up to the present, UNS may have precedence for enabling organisms to adapt to an ever-changing environment. We propose that UNS can be reused and manipulated by neocortical mechanisms, and jointly, novel skills can be developed during evolution. In a bigger conceptual framework, our findings and others, call for a significant shift from the modal dichotomous view of the exclusive role of the cortex in high-level cognition and transfer processes, and we suggest an interplay model between subcortical and cortical brain mechanisms.

## Methods

### Participants

Eighty-three students participated in two experiments for payment or course credit: 33 in Experiment 1 (mean age 22.94; 30 females); 50 in Experiment 2 (mean age 24.98; 44 females). In both experiments, all participants had normal or corrected-to-normal vision and no reported history of attention deficit or learning disabilities. Accuracy rates and average RTs of correct responses in each task were calculated. Participants were excluded from the analyses of each task if they: had performed worse than chance level (less or equal to 50% accuracy; less than 5% of all participants); or had pre-training monocular advantage (less or equal to 25% accuracy difference in performance between the eyes; less than 5% of all participants), since it may indicate a-prior monocular asymmetry that will hamper the ability to detect a monocular benefit in the post-training stage. Trials in which RTs were very fast (RT < 100 ms) or very slow (RT > 3000 ms), were excluded from the analysis (less than 5% of all trials). Studies that employed similar figural-spatial training method used sample size of 25–28 participants in each experiment^32, 33^. Because no previous study assessed the interaction with the current eye-of-origin manipulation, we used bigger sample size in each experiment (33 or 50 participants). The study was approved by the University of Haifa ethics committee and the experiments were performed in accordance with relevant guidelines and regulations. Informed consent was obtained from all participants. For pre-registration of Experiment 2 see—AsPredicted #8477, titled 'Transfer_16_2_18'.

### Stimuli and experimental design

In both experiments, during the pre-training stage, participants performed a subtraction task and the Stroop task in two separate blocks (one block for each task). The eye of presentation was randomized throughout the block, and in each trial the visual stimulus was presented to only one eye (either left or right). During the visuospatial training stage, participants performed the figural-spatial task, that was presented to only one eye in each participant (randomly and counterbalanced between the right eye and the left eye in a between-participants design), and feedback was given after each trial. In the post-training stage, participants performed subtraction, figural-spatial and the Stroop tasks. Similar to the pre-training stage, the visual stimuli were presented to only one eye and the order of eye presentation was randomized. Importantly, in the post-training tasks, the problems in arithmetic and figural-spatial tasks were the same ("old problems") or different ("novel problems") than those presented before this stage. Based on previous studies^32, 33^, during training 220 trials of figural-spatial problems were presented (10 figures * 22 repetitions each; in 22 blocks). In each problem, a drawing of a three-dimensional geometric object was presented to only one eye. Participants were required to determine the number of object faces as fast and accurately as possible. The figural-spatial problems were developed using a figural-spatial subscale of a well-established intelligence test that strongly relies on spatial visualization abilities and is included in a psychometric test^47^. Each face was defined by salient angles and can be plane, concave, or convex. For example, a cube consists of six faces whereas a sphere has only one face. The participants had two potential solutions that were presented on the same slide as the figural-spatial problem. They were instructed to press the "N" button if the number that appeared on the right side is the number of object faces or the "C" button if the number that appeared on the left side is the number of object faces. Responses were made using a QWERTY keyboard. After each trial the participants got a feedback on the answer (correct or incorrect response). Pre-training and post-training tasks were presented in a reduced Latin-square block design. We assessed the following abilities:

#### Figural-spatial abilities

As in the training stage, each experimental trial began with a fixation rectangle presented for 1 s to both eyes and afterwards each problem was presented to one eye for 5 s or until response. In the post-training, half (40 trials; ten figural-spatial stimuli presented four times each) of the problems was identical to the problems in the training ("old problems") and half (40 trials; ten figural-spatial stimuli presented four times each) was different ("novel problems"), and they were presented in a random order to each eye separately (total of 80 trials).

#### Subtraction arithmetic abilities

In each problem, participants were requested to perform a verification task, in which subtraction problems with three single-digits and their solution were presented. The participants were instructed to press the "G" button if the equation is correct and the "S" button if the equation is incorrect as fast and as accurate as they can. Before training, 16 problems were presented to each eye (total of 32 problems). In the post-training, half (2*16 problems) of the equations was identical to the equations of the pre-training tasks ("old equations") and half (2*16 problems) was different ("novel equations"), and they were presented in a random order to each eye separately (total of 64 trials). Half of the equations were correct, and half were incorrect. Each experimental trial began with a fixation rectangle appearing for 1 s to both eyes. Afterwards, a three single-digit equation and its solution were presented to one eye for 3 s or until response.

#### Executive functions abilities

Participants performed a Stroop task, in which they were presented with a word stimulus (i.e., GREEN, RED in Hebrew) in between two peripheral color patches (i.e., red, green). Participants were required to respond manually according to the color patches (press the “9” button for the red color and the “7” button for the green color), while ignoring the word meaning as fast and accurately as possible. In congruent trials, both color patches and word meaning are the same (e.g., red patches and the word RED), and in incongruent trials they differ (e.g., red patches and the word GREEN). The congruency effect (incongruent condition minus congruent condition) was computed for each participant. Before and after training, 16 trials of incongruent condition and 16 trials of congruent condition were presented randomly to each eye (32 trials were presented to the left eye and 32 trials to the right eye—total of 64 trials (16*2*2)). Each experimental trial began with a fixation rectangle appearing for 1 s to both eyes. Afterwards, the word stimulus and the two peripheral color patches were presented to one eye for 3 s or until response.

In experiment 2, in addition to all other tasks, multiplication arithmetic abilities were also assessed in the pre and post training stages. The multiplication equations were similar to the subtraction equations with the following exception: The equations contained 2 digits and a solution (e.g., 8 × 6 = 42).

## Supplementary Information

Supplementary Information.
