# Publisher Correction: Primitive visual channels have a causal role in cognitive transfer

**DOI:** 10.1038/s41598-021-95662-8

**Published:** 2021-08-06

**Authors:** William Saban, Gal Raz, Roland H. Grabner, Shai Gabay, Roi Cohen Kadosh

**Affiliations:** 1 Department of Psychology, IIPDM, University of Haifa, Haifa, Israel; 2 Department of Experimental Psychology, Wellcome Centre for Integrative Neuroimaging, University of Oxford, Oxford, UK; 3 Institute of Psychology, University of Graz, Graz, Austria; 4 Department of Psychology, Helen Wills Neuroscience Institute, University of California, Berkeley, CA USA

Correction to: *Scientific Reports* 10.1038/s41598-021-88271-y, published online 22 April 2021

In the original version of this article Shai Gabay and Roi Cohen Kadosh were omitted as equally contributing authors.

The original article has been corrected.

