# Supplementary material for: Primitive visual channels have a causal role in cognitive transfer
Source: Sci Rep. 2021 Apr 22;11:8759. doi: 10.1038/s41598-021-88271-y (PMC8062541; doi:10.1038/s41598-021-88271-y)

Supplementary Information for

Primitive Visual Channels Have a Causal Role in Cognitive Transfer

**Authors:**

William Saban*^1^, Gal Raz^2^, Roland H. Grabner^3^, Shai Gabay*^#1^, and Roi Cohen Kadosh*^#2^

**Affiliations**

1 Department of Psychology and IIPDM, University of Haifa, Israel.

2 Wellcome Centre for Integrative Neuroimaging, Department of Experimental Psychology, University of Oxford.

3 Institute of Psychology, University of Graz, Austria.

4 Helen Wills Neuroscience Institute and the Department of Psychology, University of California, Berkeley, CA, USA.

# Equally contributed.

*Correspondence to: [williamsaban@gmail.com](mailto:williamsaban@gmail.com), [shaigaba@gmail.com](mailto:shaigaba@gmail.com), and [roi.cohenkadosh@psy.ox.ac.uk](mailto:roi.cohenkadosh@psy.ox.ac.uk)

**This PDF file includes:**

Figures S1 to S2.

Link to Table S1.

Fig. S1. (A) A typical trained-eye condition in which the arithmetic equations (subtraction) is presented to the right eye (right column) and the figural – spatial training is also presented to the right eye (right column). The middle column represents the participant's fused perception. (B) A typical untrained-eye condition in which the arithmetic equations (subtraction) is presented to the right eye (right column) and the figural – spatial training is presented to the left eye (left column). The middle column represents the participant's fused perception. Note, in both Eye conditions the percept is the same.


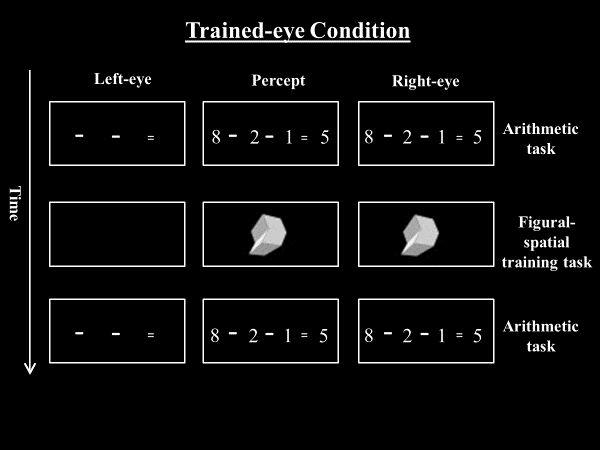


A


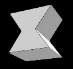

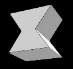

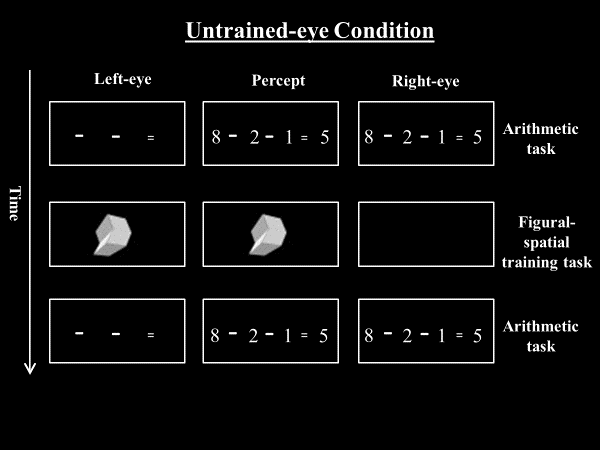

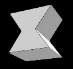

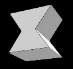


B

[**Table S1**](https://docs.google.com/spreadsheets/d/e/2PACX-1vQKoSZ6IK2kh606UpB_aj6hF0DTv27dC6cn8zn6EDDf3iqUVzwsVQ6lesMWv4PEpCwAkBwXvoM_dXEC/pubhtml)**:**

|  |  | **Experiment 1** |  | **Experiment 2** |  |
| --- | --- | --- | --- | --- | --- |
|  |  | **RT** | **Accuracy** | **RT** | **Accuracy** |
| **Task** | **Effect** |  |  |  |  |
| **Figural-spatial task** |  |  |  |  |  |
|  | **Eye** | *p*= .73; F(1,3)=.125; ηp2< .01 | *p*=.060; F(1,31)=3.81; ηp2=.11 | *p*=.009; F(1,46)=7.49; ηp2=.14 | *p*=.92; F(1,46)=.01; ηp2<.01 |
|  | **Problems-novelty** | *p*<.001; F(1,31)=205.2; ηp2=.87 | *p*<.001; F(1,31)=23.31; ηp2=.41 | *p*<.001; F(1,46)=96.92; ηp2=.68 | *p*<.001; F(1,46)=21.56; ηp2=.32 |
|  | **Eye*problems-novelty** | *p*=.80; F(1,31)=.065; ηp2<.01 | *p*=.008; F(1,31)=8.02; ηp2=.21 | *p*=.029; F(1,46)=5.06; ηp2=.09 | *p*=.54; F(1,46)=.38; ηp2<.01 |
| **Subtraction Old** |  |  |  |  |  |
|  | **Eye** | *p*=.16; F(1,28)=2.07; ηp2=.06 | *p*=.4; F(1,28)=.73; ηp2=.02 | *p*=.84; F(1,45)=.04; ηp2<.01 | *p*=.92; F(1,45)=.01; ηp2<.01 |
|  | **Experimental stage** | *p*<.001; F(1,28)=49.85; ηp2=.64 | *p*=.006; F(1,28)=8.66; ηp2=.236 | *p*<.001; F(1,45)=36.91; ηp2=.45 | *p*=.001; F(1,45)=12.3; ηp2=.21 |
|  | **Eye*Experimental stage** | *p*=.73; F(1,28)=.12; ηp2<.01 | *p*=.173; F(1,28)=1.96; ηp2=.06 | *p*=.77; F(1,45)=.09; ηp2<.01 | *p*=.74; F(1,45)=.11; ηp2<.01 |
| **Subtraction Novel** |  |  |  |  |  |
|  | **Eye** | *p*=.54; F(1,28)=.39; ηp2=.01 | *p*=.026; F(1,28)=5.50; ηp2=.16 | *p*=.70; F(1,45)=.15; ηp2<.01 | *p*=.023; F(1,45)=5.53; ηp2=.11 |
|  | **Experimental stage** | *p*<.001; F(1,28)=32.68; ηp2=.54 | *p*<.001; F(1,28)=13.29; ηp2=.32 | *p*<.001; F(1,45)=31.68; ηp2=.41 | *p*=.017; F(1,45)=6.15; ηp2=.12 |
|  | **Eye*Experimental stage** | *p*=.274; F(1,28)=1.40; ηp2=.04 | *p*=.008; F(1,28)=8.14; ηp2=.22 | *p*=.32; F(1,45)=1.03; ηp2=.02 | *p*=.045; F(1,45)=4.24; ηp2=.09 |
| **Stroop task** |  |  |  |  |  |
|  | **Eye** | *p*=.73; F(1,32)=3.431; ηp2=.09 | *p*=.006; F(1,32)=8.79; ηp2=.215 | *p*=.46; F(1,41)=.55; ηp2<.01 | *p*=.51; F(1,41)=.43; ηp2=.01 |
|  | **Experimental stage** | *p*=.67; F(1,32)=.188; ηp2<.01 | *p*=.36; F(1,32)=.88; ηp2ηp2=.027 | *p*=.32; F(1,41)=1.00; ηp2<.01 | *p*=.81; F(1,41)=.06; ηp2<.01 |
|  | **Eye*Experimental stage** | *p*=.79; F(1,32)=.074; ηp2<.01 | *p*=.39; F(1,32)=.75; ηp2=.023 | *p*=.43; F(1,41)=.63; ηp2=.01 | *p*=.88; F(1,41)=.02; ηp2<.01 |
| **Multiplication Old** |  |  |  |  |  |
|  | **Eye** |  |  | *p*=.86; F(1,46)=.03; ηp2<.01 | *p*=.38; F(1,46)=.79; ηp2<.01 |
|  | **Experimental stage** |  |  | *p*<.001; F(1,46)=31.00; ηp2=.40 | *p*=1; F(1,46)=.00 ; ηp2<.01 |
|  | **Eye*Experimental stage** |  |  | *p*=1; F(1,46)=.00 ; ηp2<.01 | *p*=.63; F(1,46)=.24; ηp2<.01 |
| **Multiplication Novel** |  |  |  |  |  |
|  | **Eye** |  |  | *p*=.84; F(1,46)=.04; ηp2<.01 | *p*=.61; F(1,46)=.27; ηp2<.01 |
|  | **Experimental stage** |  |  | *p*=.004; F(1,46)=8.97; ηp2=.16 | *p*=.94; F(1,46)=.005; ηp2<.01 |
|  | **Eye*Experimental stage** |  |  | *p*=1; F(1,46)=.00 ; ηp2<.01 | *p*=.82; F(1,46)=.05; ηp2<.01 |
| ηp2=effect size |  |  |  |  |  |

Fig. S2. Learning curve during training in each experiment.


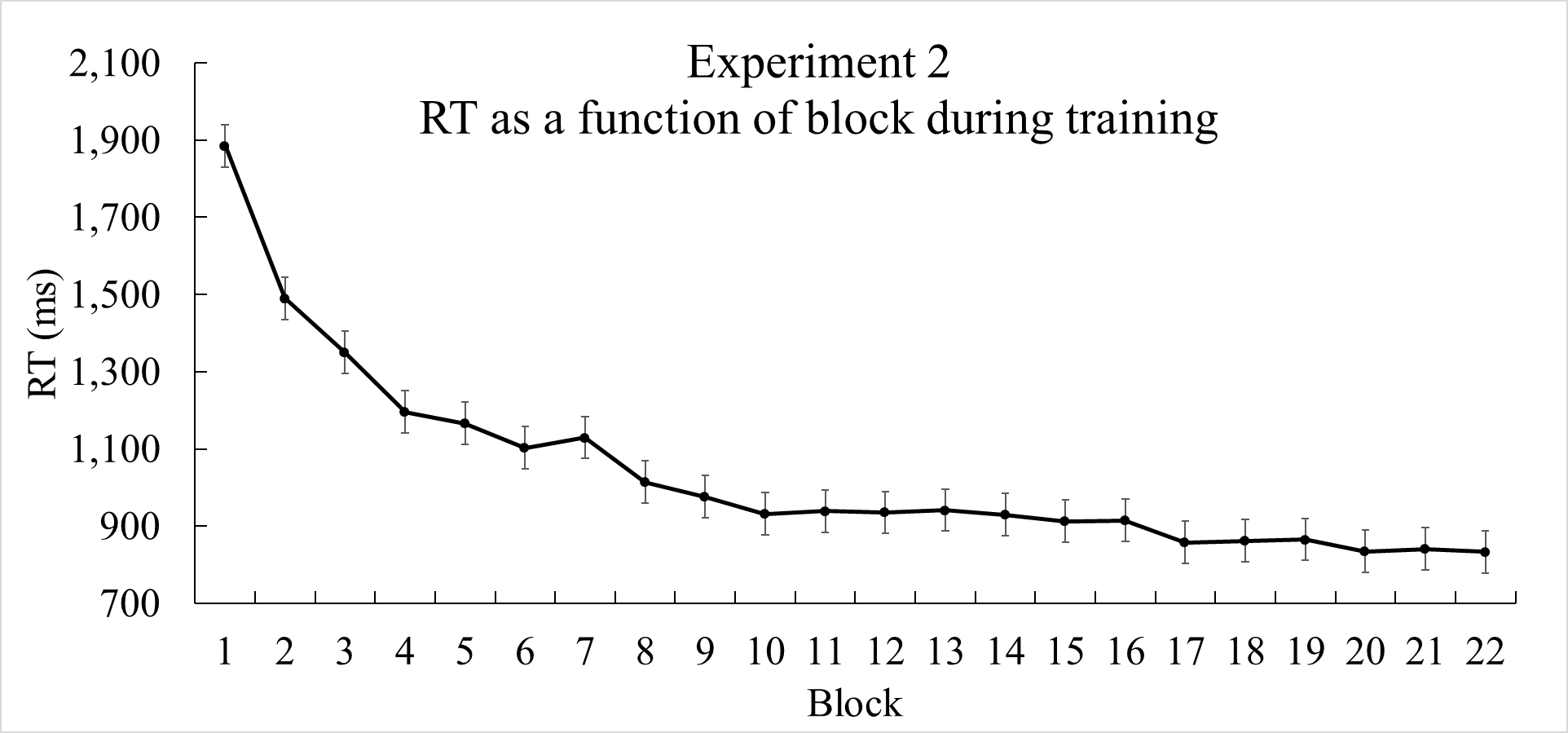

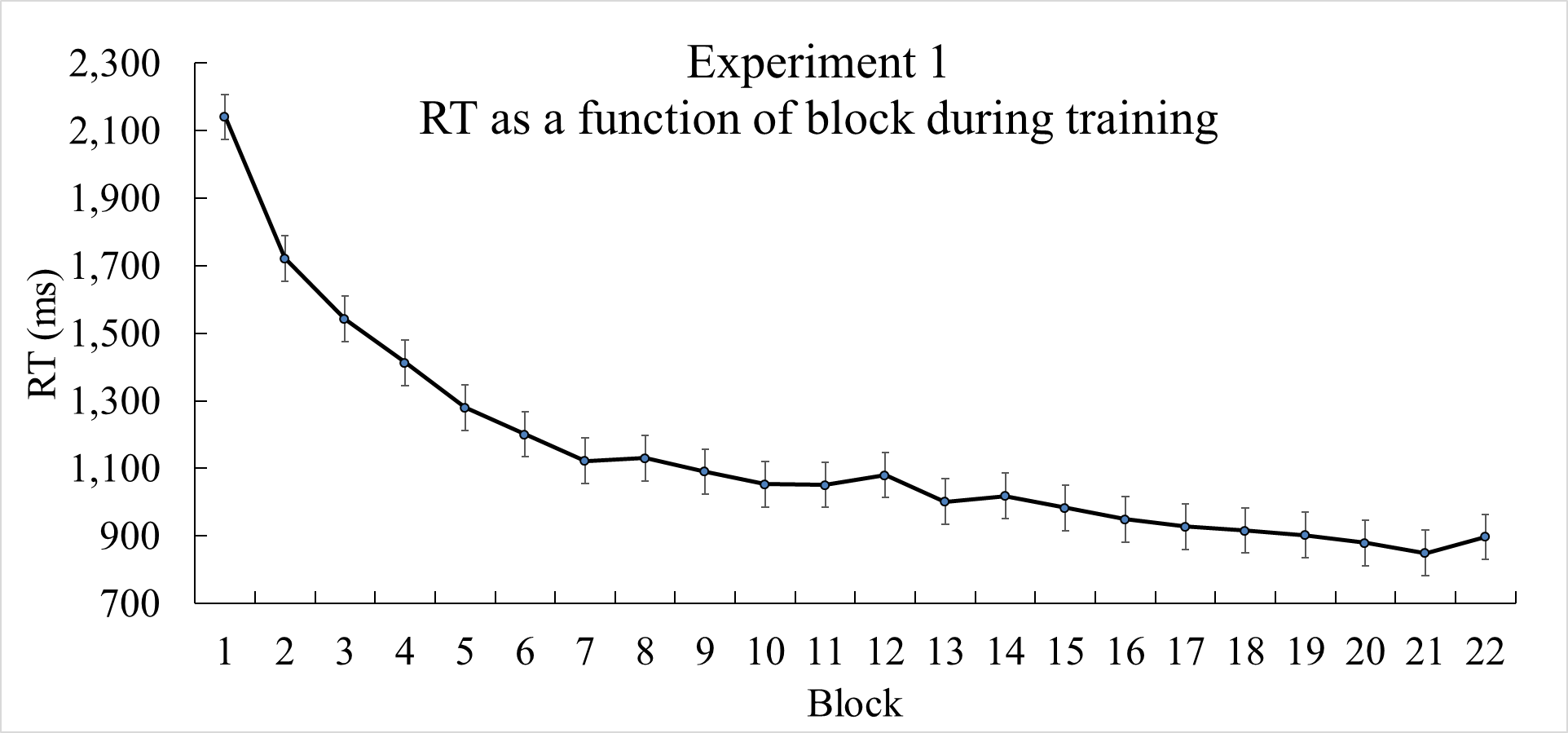

Supplement: Supplementary file 1 — Supplementary Information. [file 41598_2021_88271_MOESM1_ESM.docx]
